# Glasgow Coma Scale and Outcomes after Structural Traumatic Head Injury in Early Childhood

**DOI:** 10.1371/journal.pone.0082245

**Published:** 2013-12-02

**Authors:** Natasha L. Heather, José G. B. Derraik, John Beca, Paul L. Hofman, Rangi Dansey, James Hamill, Wayne S. Cutfield

**Affiliations:** 1 Liggins Institute, University of Auckland, Auckland, New Zealand; 2 National Research Centre for Growth and Development, University of Auckland, Auckland, New Zealand; 3 Paediatric Intensive Care Unit, Starship Children’s Hospital, Auckland District Health Board, Auckland, New Zealand; 4 Trauma Service, Starship Children’s Hospital, Auckland District Health Board, Auckland, New Zealand; University of Pittsburgh, United States of America

## Abstract

**Objective:**

To assess the association of the Glasgow Coma Scale (GCS) with radiological evidence of head injury (the Abbreviated Injury Scale for the head region, AIS-HR) in young children hospitalized with traumatic head injury (THI), and the predictive value of GCS and AIS-HR scores for long-term impairment.

**Methods:**

Our study involved a 10-year retrospective review of a database encompassing all patients admitted to Starship Children’s Hospital (Auckland, New Zealand, 2000–2010) with THI.

**Results:**

We studied 619 children aged <5 years at the time of THI, with long-term outcome data available for 161 subjects. Both GCS and AIS-HR scores were predictive of length of intensive care unit and hospital stay (all p<0.001). GCS was correlated with AIS-HR (ρ=-0.46; p<0.001), although mild GCS scores (13–15) commonly under-estimated the severity of radiological injury: 42% of children with mild GCS scores had serious–critical THI (AIS-HR 3–5). Increasingly severe GCS or AIS-HR scores were both associated with a greater likelihood of long-term impairment (neurological disability, residual problems, and educational support). However, long-term impairment was also relatively common in children with mild GCS scores paired with structural THI more severe than a simple linear skull fracture.

**Conclusion:**

Severe GCS scores will identify most cases of severe radiological injury in early childhood, and are good predictors of poor long-term outcome. However, young children admitted to hospital with structural THI and mild GCS scores have an appreciable risk of long-term disability, and also warrant long-term follow-up.

## Introduction

Traumatic head injury (THI, defined here as injury to the scalp, skull or brain) is a very common childhood event [1], and brain injuries are the most frequent cause of trauma fatality during childhood [2]. The Glasgow Coma Scale (GCS) is a simple tool used to assess level of consciousness following head injuries, with lower scores denoting greater impairment and more severe THI [3]. However, GCS scores are subject to a number of limitations, including inter-observer reliability [4-6], time elapsed since injury, as well as the confounding effects of seizures, early sedation or intubation, and physiological shock [7]. Furthermore, the assessment of neurological status can be particularly challenging in very young, pre-verbal children [8]. The GCS is designed for an adult level of cognition, but modified versions are typically used to assess young children. 

Given their inherent limitations, it is not surprising that GCS scores show a weak and inconsistent association with survival, functional outcome, and radiological severity scores within adult THI populations [9-13]. During childhood, lower GCS scores predict a greater likelihood of intra-cranial injury and poor outcome following THI [14-18]. However, few studies report acute injury data that are specific to early childhood [17]. Further, there is a weak relationship between injury severity according to initial GCS score and long-term cognitive and behavioural outcomes following early childhood THI [19,20]. 

THI can also be graded in terms of radiological markers of injury, which may provide a more reliable index of severity than GCS scores in early childhood. The Abbreviated Injury Scale (AIS) is an anatomical system for scoring traumatic injury. Injuries within defined body regions are ranked on a “threat to life” scale of 1 to 6 (1 – mild, 2 – moderate, 3 – serious, 4 – severe, 5 – critical, and 6 – not survivable) [21]. AIS scores for the head region (AIS-HR) are predominantly based on computerised tomography (CT) findings, and correlate with both mortality and functional outcome in adult populations [9-11]. Examples of AIS-HR scores for specific head injuries are given in Table S1. Where more than one injury is present within an anatomical region, the region is scored according to the most severe injury. Recent versions of the AIS include consensus-derived adaptations for childhood, although these have not been extensively validated [21]. In particular, there are no previous data looking at the predictive value of AIS-HR scores in early childhood.

This study aimed to assess the predictive value of post-resuscitation GCS on short- and long-term outcomes in young children admitted to a tertiary paediatric hospital with THI. We examined the association between post-resuscitation GCS, AIS-HR, and short-term markers of injury severity (including length of intensive care unit and hospital admission) after early childhood THI. In addition, we assessed the association between GCS and AIS-HR scores with disability in patients with follow-up data.

## Methods

### Ethics Statement

Ethics approval for this study was provided by the Northern X Regional Ethics Committee (Ministry of Health, New Zealand) and the Auckland District Health Board Research Review Committee. For follow-up participants, written informed consent was obtained from parents or guardians, as well as verbal or written consent from each child as was appropriate to their age.

### Study design

Starship Children’s Hospital is the only paediatric neurosurgical centre in the greater Auckland area (New Zealand), receiving all children admitted with THI in the region (population approximately 1.5 million). Patients admitted with THI at an age <5 years were identified from the Starship Children’s Hospital Trauma database (which includes information on all inpatient admissions with childhood THI) over a 10-year period from 2000–2010. This study excluded children who died during the acute admission or whose primary injury was extra-cranial (e.g. major abdominal trauma or limb fracture) paired with relatively mild THI, as their inclusion could distort ward and ICU admission data. 

Demographic data included age at injury, gender, survival to discharge, and duration of admission to both ICU and hospital. GCS score was obtained from emergency department admission forms, and represented the first formal post-resuscitation score reported in the notes, as is common practice in TBI studies [9]. Our institution uses a modified verbal component to the GCS for children aged less than two years, such that a verbal score of 5 describes a best response of smiling, cooing, or appropriate crying, 4 describes consolable crying, 3 describes inconsolable crying or irritability, 2 describes grunting or agitation, and 1 no response. The motor component is similarly modified, such that patients are allocated the highest score of 6 for normal spontaneous movement rather than obeying commands. GCS scores were classified as mild (GCS 13–15), moderate (9–12), or severe (3–8) [22]. In addition, the head region AIS (1990 version) was scored based on the initial computerised tomography (CT) scan [21]. As a result, our analyses only included children who had undergone an acute CT head scan, which enabled determination of AIS-HR scores.

Within our institution, the absolute indications for performing an acute CT head scan include GCS score ≤13, neurological deterioration, focal neurological signs, penetrating injury, or a depressed skull fracture. Relative indications include high risk injury mechanism, ≥4 episodes of vomiting, loss of consciousness >1 minute, prolonged lethargy or irritability, or infants with a scalp haematoma. Children with structural THI evident on CT scan (excluding simple linear skull fracture) are routinely admitted to hospital, as are children with delayed seizures, disabling symptoms, poor access to medical care, and cases of suspected child abuse. 

Long-term outcome data were available for a number of patients, who had previously been recruited for follow-up as part of another THI study [23]. Further selection criteria included structural THI with head region AIS-HR ≥2 (essentially a skull fracture, intracranial haemorrhage, or cerebral injury), living within the greater Auckland region at the time of assessment, and a minimum interval of 12 months between THI and assessment. 

All follow-up clinical assessments were carried out in person at the Maurice & Agnes Paykel Clinical Research Unit (Liggins Institute, University of Auckland) by interviewers who were blinded to injury details. Assessments included neurological examination and enquiry about visual or hearing impairment. Neurological disability was defined as motor or sensory deficit sufficient to impair function. In addition, parents were asked about epilepsy, changes in behaviour, as well as special education needs and input. The child’s overall functional status was assessed according to the King’s Outcome Scale for Childhood Head Injury (KOSCHI) [24], a paediatric adaptation of the original Glasgow Outcome Scale. The KOSCHI scale incorporates social and behavioural problems following THI [24], and so has greater sensitivity for functional disability. Assessments were based on parental report and supported by clinical findings. THI outcomes were either classified as good (score of 5, no sequela that impact on well-being or function) or those including residual problems (4 – moderate disability, 3 – severe disability, 2 – vegetative state) [24]. 

For participants who were followed up, data on ethnicity and socio-economic status were also recorded. Ethnicity was identified by self-report using a prioritised system, such that if multiple ethnicities were selected, the subject was assigned to a single ethnicity, based on a hierarchical classification [25]. Socio-economic status was determined using geo-coded deprivation scores derived from current address, using the New Zealand Index of Deprivation 2006 (NZDep2006) [26].

### Statistical analyses

Non-parametric Spearman’s rank correlations were used to examine the association between GCS and AIS-HR scores, as well as to compare the association between each scale with length of hospitalization and ICU stay. For multivariate models, GCS scores were categorized as previously described (mild, moderate, and severe), while AIS-HR scores were categorized as mild–moderate (AIS-HR 1–2), serious–severe (AIS-HR 3–4), and critical (AIS-HR 5). Binary logistic regressions were used to assess the effect of GCS or AIS-HR categories on the likelihood of children staying in hospital ≥48 hours and being admitted to ICU. Confounding factors were also included in the models, namely ethnicity and sex as categorical variables, and age at injury as a continuous variable. 

Baseline data for follow-up participants were compared to the remaining patients in the large cohort using one-way ANOVA, the non-parametric Kruskal-Wallis test, or the 2-sample Poisson rate test. For the follow-up data, binary logistic regressions were adopted to evaluate the association of GCS or AIS-HR categories with sub-normal KOSCHI outcomes, long-term neurological disability, and allocation of teacher aide at school. Confounding factors included in the models were ethnicity and sex as categorical variables, as well as age at injury, time interval from injury to assessment, and NZDep2006 scores as continuous variables. 

Statistical analyses were carried out in SAS v.9.2 (SAS Institute, Cary, NC, USA) and Minitab v.16 (Pennsylvania State University, USA). The c statistic (corresponding to the area under the receiver operating characteristic (ROC) curve) is provided for binary logistic regressions to indicate the predictive ability of individual models. All statistical tests were two-tailed and maintained at a 5% significance level.

## Results

**Figure 1:**
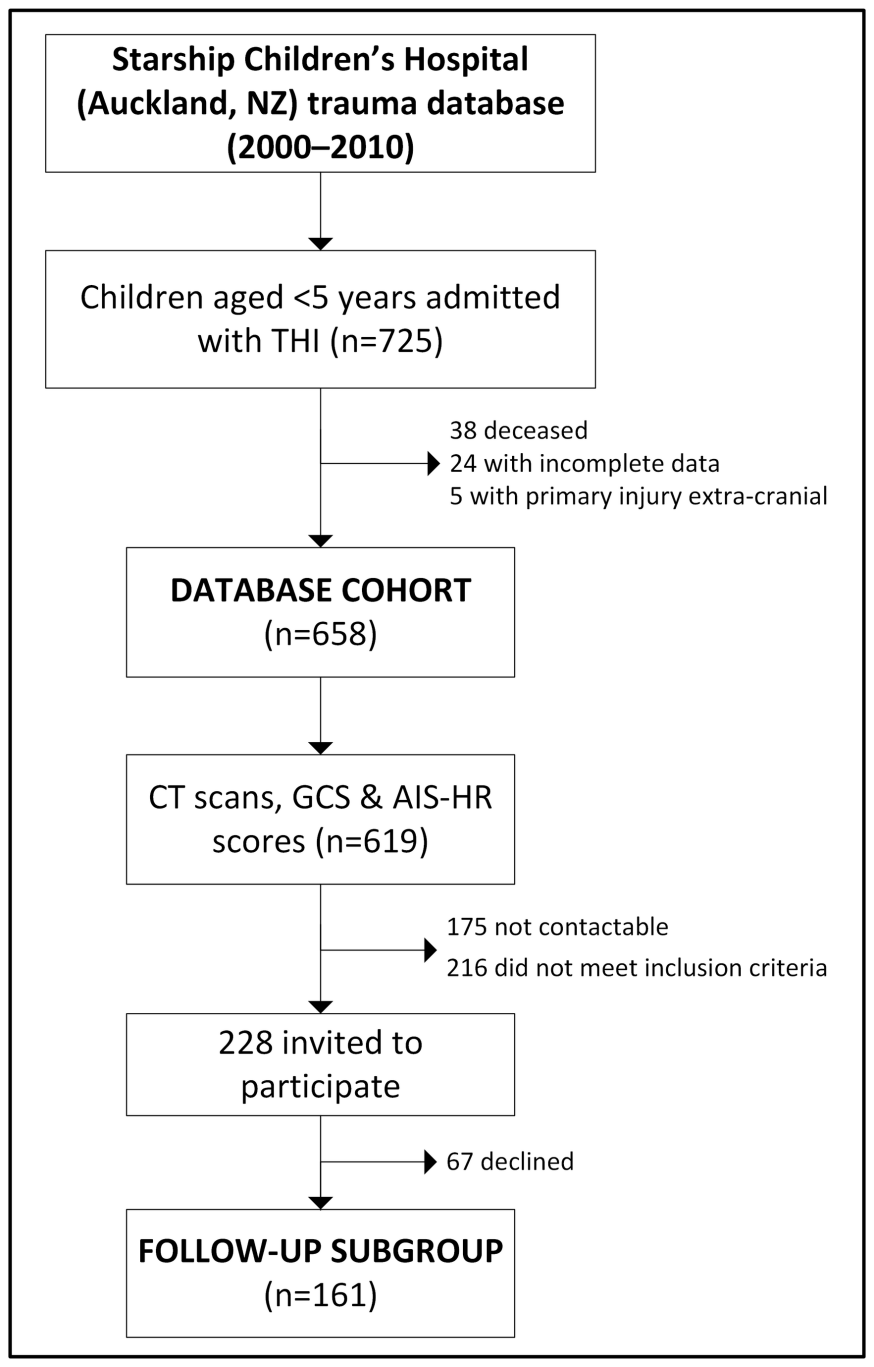
Summary of study's recruitment.

725 children with THI who were less than five years of age were admitted to Starship Children’s Hospital over the 10-year study period (Figure 1). 38 children died (5%) during the admission (all of whom had severe GCS and severe–critical AIS-HR scores), and another 29 cases were excluded (24 due to incomplete data and 5 whose primary injury was extra-cranial) (Figure 1). Of the remaining 658 THI cases, 619 children had CT scans and were ascribed both GCS and AIS scores at the time of hospital admission (Figure 1), of which 176 (28%) required admission to ICU. Baseline characteristics are shown in Table 1. 

**Table 1:** Demography of the studied population.

		**THI database**	**Follow-up participants**	**Cohort remainder**
**n**		619	161	458
**Age at injury (years)**		1.9 ± 1.5	2.0 ± 1.6	1.9 ± 1.5
**Sex ratio (% boys)**		63	60	65
**Age at follow-up assessment (years)**		-	7.7 ± 2.6	-
**Time from injury to follow-up (years)**		-	5.7 ± 2.3	-
**GCS scores**	**Median score (IQR)**	15 (12–15)	13 (8–15)^***^	15 (13–15)
	**Mild (13–15)**	71%	53%^***^	77%
	**Moderate (9–12)**	13%	21%^***^	10%
	**Severe (3–8)**	16%	26%^***^	13%
**AIS-HR scores**	**Median score (IQR)**	3 (2–4)	4 (3–4)^***^	2 (2–4)
	**Mild–moderate (1-2)**	42%	15%^***^	52%
	**Serious–severe (3-4)**	48%	70%^***^	40%
	**Critical (5)**	10%	15%^**^	8%
**Days in hospital (median (IQR))**		2 (1–8)	5 (1–13)^***^	2 (1–6)
**Intensive care unit admission**		28%	41%^***^	24%
**Days in ICU (median (IQR))**		2 (1–7)	3 (1–10)^*^	2 (1–5)

Age-related data are means ± SD. ^*^p<0.05, ^**^p<0.01, ^***^p<0.001 for follow-up participants vs the cohort remainder. Length of intensive care unit (ICU) stay refers solely to the population that was admitted to ICU. IQR – inter-quartile range.

### GCS versus AIS-HR

**Figure 2:**
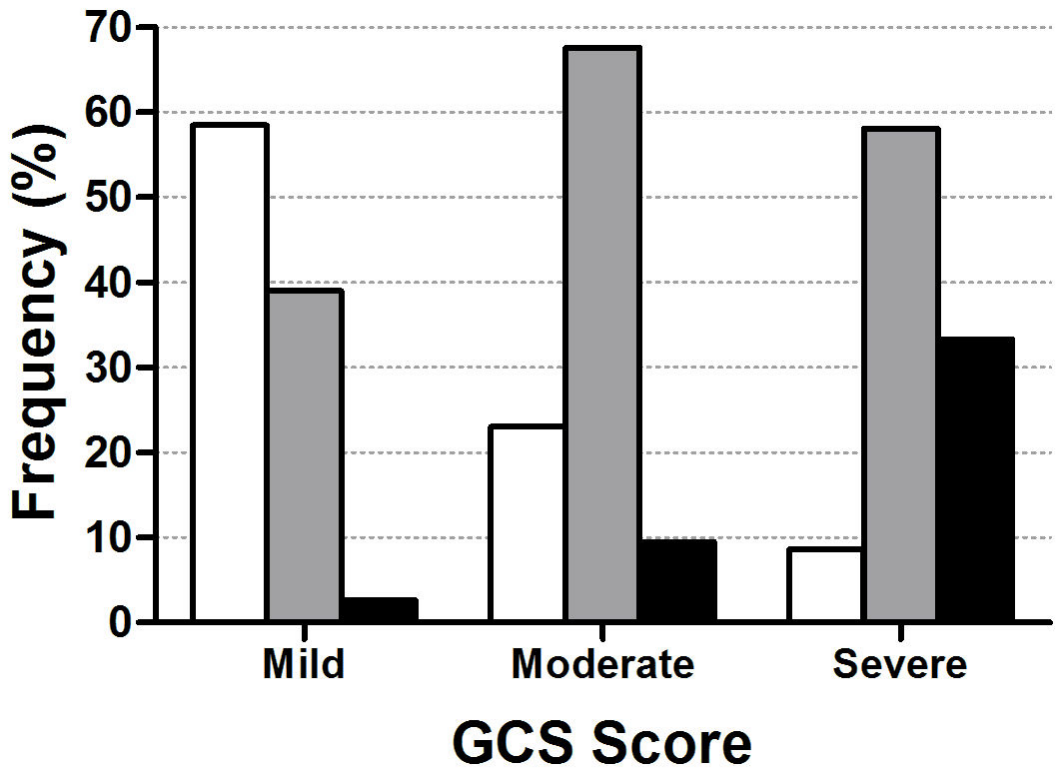
The association between GCS and AIS-HR scores among children who suffered early childhood traumatic head injury. Bars represent AIS-HR scores, so that the darker the bar the more serious the radiological injury: mild–moderate (white), serious–severe (gray), and critical (black) (n=594). Overall, the two scales were associated as per non-parametric Spearman’s rank correlations (ρ=-0.46; p<0.001).

GCS and AIS-HR scores were correlated (ρ=-0.46; p<0.001), and the relationship between the two scales is illustrated in Figure 2. Within our cohort of children admitted to hospital with THI, severe GCS was a good predictor of severe radiological injury, however mild GCS scores were less predictive of mild radiological injury (Figure 2). Thus, 39% of children with mild GCS had serious–severe radiological injury (AIS-HR 3 or 4), and a further 3% had critical THI (AIS-HR 5; Figure 1). Importantly, the child’s age at THI appeared to have little effect on this association, as the relationship between GCS and AIS-HR scores was very similar among children <2 and 2–5 years of age (data not shown). 

### Stay in hospital and ICU

Both GCS and AIS-HR scores were highly predictive of hospitalization for >48 hours and ICU admission (Table 2). Overall, GCS scores were stronger predictors of the length of ICU admission (ρ=-0.70; p<0.001) than AIS-HR (ρ=0.50; p<0.001). However, the association between GCS and AIS-HR with duration of hospital admission was virtually identical (GCS ρ=-0.59, p<0.001; AIS-HR ρ=0.59; p<0.001). Neither sex nor age at injury was associated with length of admission to hospital or ICU, when AIS-HR or GCS scores were accounted for. Note that separate analyses of the data for children <2 and 2–5 years yielded similar results (data not shown).

**Table 2:** Hospitalization and intensive care unit (ICU) admission among children admitted to hospital with traumatic head injury (n=594).

		**Hospitalization >48 hr**	**ICU admission**
**n**		307	176
**AIS-HR scores**	**Mild–moderate (1-2)**	1.0 (reference)^***^	1.0 (reference)^***^
	**Serious–severe (3-4)**	6.7 (4.5–10.0)	6.1 (3.8–9.9)
	**Critical (5)**	143 (8.6–1000)	84.9 (31.7–227)
	**c statistic**	0.77	0.78
**GCS scores**	**Mild (13-15)**	1.0 (reference)^***^	1.0 (reference)^***^
	**Moderate (9-12)**	5.9 (3.1–11.2)	11.5 (6.7–19.7)
	**Severe (3-8)**	66.7 (13.1–339)	127 (56.2–286)
	**c statistic**	0.71	0.88

Results are from logistic regression models, and data are presented as odds ratios and 95% confidence intervals according to Abbreviated Injury Scale scores for the head region (AIS-HR) and Glasgow Coma Scale (GCS) scores. ^***^p<0.001 for the association of AIS-HR or GCS with the outcome measure. The c statistic corresponds to the area under the receiver operating characteristic (ROC) curve, and it varies between 0.5 and 1.0, with higher values indicating higher predictive ability.

### Long-term outcomes

From the 619 subjects who had CT scans, 65 had less than the minimum one-year follow-up period, 109 had an AIS-HR score of 1, 42 were identified as not living within the greater Auckland region, and 175 were not contactable (Figure 1). As a result, 228 families were invited to participate in the follow-up study but 67 declined, so that follow-up data were available for 161 children (Figure 1). These children suffered comparatively more severe injuries than the other subjects in the database. They stayed longer in hospital (p<0.001), had a greater frequency of admissions to ICU (p<0.001), and encompassed more subjects with moderate (p<0.001) and severe (p<0.001) GCS scores, as well as serious–severe (p<0.001) and critical (p<0.01) AIS-HR scores compared to the remainder of subjects in the larger cohort (Table 1). 

At follow-up, 98 (61%) of children had made a complete recovery by KOSCHI rating (score of 5), whilst 56 had residual problems that were graded as moderate (38%, KOSCHI 4) and 7 as severe (4%, KOSCHI 3). Overall, 39 children (24%) had motor or sensory neurological deficits. Individual subjects often had multiple neurological deficits. There were 18 subjects with impaired vision (cortical blindness, visual field defect, or paralytic strabismus), six with sensorineural hearing loss, and 30 with motor weakness (predominantly hemiplegia). Seven children (4%) were educated in a special needs class, while a further 36 (22%) were allocated time with an individual teacher aide because of intellectual (rather than physical) disability. There was considerable overlap between those children with neurological deficits and those requiring educational support, with 25 of the 36 children allocated a teacher aide also having a neurological deficit. In addition, nine subjects were diagnosed with epilepsy, and six with attention deficit hyperactivity disorder. 

Both GCS (ρ=0.44; p<0.001) and AIS-HR (ρ=0.36; p<0.001) were correlated with KOSCHI scores. As a result, multivariate analyses showed that children with a severe GCS score or a critical AIS-HR score were considerably more likely to have residual problems (as per KOSCHI scores) at follow-up (Table 3). GCS and AIS-HR scores were also good predictors of neurological disability, whose odds were much greater among children with severe GCS scores or a critical radiological injury (Table 3). Further, among school-aged children (≥5 years; n=131), GCS and AIS-HR scores were also predictive of the need for educational support in school (Table 3). Notably, long-term outcome measures were largely unaffected by sex, ethnicity, socio-economic status (NZDep2006), age at injury, or interval between injury and assessment.

**Table 3:** Adverse long-term outcomes among 161 children admitted to hospital with traumatic head injury.

		**Neurological disability**	**Residual problems**	**Educational support**
**AIS-HR scores**	**Mild–moderate (1-2)**	1.0 (reference)^***^	1.0 (reference)^**^	1.0 (reference)^*^
	**Serious–severe (3-4)**	6.4 (0.4–97.0)	3.1 (0.8–11.7)	5.5 (1.0–30.8)
	**Critical (5)**	41.6 (2.4–715)	18.7 (3.6–98.6)	13.8 (1.9–101)
	**c statistic**	0.76	0.76	0.71
**GCS scores**	**Mild (13-15)**	1.0 (reference)^***^	1.0 (reference)^***^	1.0 (reference)^**^
	**Moderate (9-12)**	3.6 (0.6–20.5)	1.0 (0.4–2.8)	2.5 (0.4–14.6)
	**Severe (3-8)**	46.3 (8.3–258)	6.7 (2.4–19.0)	20.8 (3.8–114)
	**c statistic**	0.93	0.79	0.82

Educational support refers to the allocation of an individual teacher aide or placement in a special needs class, and the respective data covers only school-aged children (≥5 years of age; n=131). Neurological disability – sensory or motor deficit; and residual problems – score of <5 as per the King’s outcome scale for childhood head injury (KOSCHI). Results are from logistic regression models, and data are presented as odds ratios and 95% confidence intervals according to Abbreviated Injury Scale scores for the head region (AIS-HR) and Glasgow Coma Scale (GCS) scores. ^*^p<0.05, ^**^p<0.01, ^***^p<0.001 for the association of AIS-HR or GCS with the outcome measure. The c statistic corresponds to the area under the receiver operating characteristic (ROC) curve and it varies between 0.5 and 1.0, with higher values indicating higher predictive ability.

### Injury severity

There were 23 children diagnosed with radiological THI consisting solely of simple linear skull fractures (i.e. AIS-HR score of 2). Of these, 20 subjects were ascribed a mild GCS score and the remaining three a moderate score. None of these children were left with motor or sensory neurological deficits or were allocated a teacher aide. Only two children were graded as having residual problems (KOSCHI 4), with parents reporting poor concentration and specific learning difficulties in both cases. Both children had suffered short distance falls (<1.5 m) at around 18 months of age. 

Among the children who suffered THI more serious than a simple linear skull fracture, mild GCS scores were poor predictors of long-term outcome (Table 4). In this group of children, 9% of subjects had a motor or sensory neurological deficit, 18% were allocated an individual teacher aide, and 34% reported residual problems (KOSCHI 3-4, predominantly behavioural and learning difficulties). Within this subgroup, there was little overlap between the need for educational support and neurological deficits, as out of the 10 children allocated a teacher aide, only 2 had neurological deficits. Of note, the rate of adverse long-term outcomes in this group of children was similar among those ascribed mild and moderate GCS scores (Table 4). 

**Table 4:** Glasgow Coma Scale (GCS) scores and the prevalence of long-term disability among children admitted to hospital with traumatic head injury, with a radiological diagnosis more severe than a simple linear skull fracture (n=138).

**GCS Scores**	**Neurological disability**	**Residual problems**	**Educational support**
**Mild (13-15)**	9% (6/65)	34% (22/65)	18% (10/56)
**Moderate (9-12)**	23% (7/31)	35% (11/31)	22% (5/23)
**Severe (3-8)**	62% (26/42)	76% (32/42)	60% (21/35)
**All participants**	28% (39/138)	47% (65/138)	32% (36/114)

Educational support refers to the allocation of an individual teacher aide or placement in a special needs class, and the respective data covers only school-aged children (≥5 years of age; n=114). Neurological disability – sensory or motor deficit; and residual problems – score of <5 as per the King’s outcome scale for childhood head injury (KOSCHI).

## Discussion

Among young children admitted to hospital with THI, mild GCS scores commonly under-estimated the degree of structural THI. Further, follow-up of those with mild GCS scores and structural THI revealed an appreciable risk of long-term disability, particularly learning and behavioural problems. However, we did find that severe GCS scores were good predictors of both short- and long-term outcome after early childhood THI. 

The literature reports a very low prevalence of long-term cognitive deficits, and educational or behavioural problems, among children admitted to hospital with mild traumatic brain injury [27]. However, we showed that a third of young children admitted to hospital with mild GCS scores and structural THI reported on-going problems. Although three quarters of available subjects presented for long-term assessments, it is possible that subjects with ongoing concerns were more motivated to attend, so that the risk of long-term disability might have been over-estimated. Our follow-up participants were children who suffered relatively severe THI, all of whom underwent an acute CT scan, and were found to have either a skull fracture or intra-cranial injury. In this group, the prevalence of educational support and residual problems was similar in children ascribed mild or moderate GCS scores. In contrast, it is reassuring to note that the smaller sub-group of children whose worst structural diagnosis was a simple linear skull fracture did well. Such fractures are relatively common in young children with THI, and do not carry the same risk of long-term sequelae as more severe structural injuries.

Despite concerns that the GCS may be less reliable in infancy, we found that age at injury did not affect the relationship between GCS and AIS-HR scores. Nonetheless, mild GCS scores provided an unreliable estimate of structural THI severity, as a third of young children admitted to our institution with mild GCS scores had severe radiological markers of THI (i.e. severe–critical AIS-HR, indicating significant intracranial injury, such as intra-cerebral haemorrhage, or extensive cerebral contusion). No previous studies have compared AIS and GCS scores in young children, and, as our study encompassed over 600 subjects with THI and represented the 10-year experience of a tertiary paediatric hospital, the results are likely to be applicable to other populations. However, it is important to note that our analyses were based on inpatient hospital admissions with THI. Thus, our findings are not representative of the larger group of young children who present to an emergency department or family doctor with THI and mild GCS scores, who have a much lower likelihood of structural THI. Clearly, it would be inappropriate to perform CT scans in all young children with mild THI, and validated clinical prediction rules outline the indications for imaging in infants and children [28]. 

The limitations of this study include the use of retrospective data, which were taken from the trauma database of a tertiary children’s hospital. However, AIS-HR scores were assigned in a uniform manner, and were allocated by a single trained user throughout the 10-year period. The coding rules for AIS-HR specify that scores be based on imaging performed within 24 hours of injury or at first diagnosis, and are therefore almost exclusively based on CT head scans. Due to the greater sensitivity of MRI, it is possible that there would be a stronger relationship between anatomical injury on MRI scans and functional outcomes. However, MRI use would prevent injury scoring according to AIS, and the prolonged scanning time required for a head MRI scan is challenging in a child with an acute head injury.

Although GCS scores were taken from the medical notes, this was also done in a consistent manner. Follow-up assessments were cross-sectional and occurred at varied intervals after THI, but this interval was included in our multivariate models. In addition, the participants studied for long-term disability had relatively more severe injuries, and thus a greater risk of long-term sequelae than the remaining patients in the database, which may limit wider applicability of our findings. Lastly, we acknowledge that the KOSCHI outcome categories are relatively broad, and that parental report may over-estimate the impact of THI on behaviour and learning, particularly where injury occurred during infancy and there is consequently little available information on pre-injury characteristics.

Overall, our data indicate that severe GCS scores in early childhood will identify most cases of THI with severe radiological injury and requiring urgent initial management, and these scores are also good predictors of poor long-term outcome. However, it is important to recognise the limitations of GCS scores in early childhood. Among young children admitted to hospital with structural traumatic head injury, mild GCS scores did not reliably indicate a mild injury, and these children also carried an appreciable risk of long-term disability. We recommend the use of the AIS-HR in addition to GCS scores in early childhood THI. The AIS-HR is a simple tool that assists in prediciting young children at risk of ongoing disability, who warrant long-term follow-up. 

## Supporting Information

Table S1 **Summary of Abbreviated Injury Scores for the head region (AIS-HR), adapted from: Association for the Advancement of Automotive Medicine** (2001) **The** **Abbreviated** **Injury** **Scale, 1990** **Revision, Update 98**.
Barrington, IL: Association for the Advancement of Automotive Medicine.
(PDF)
