# Supplementary material for: Glasgow Coma Scale and Outcomes after Structural Traumatic Head Injury in Early Childhood
Source: PLoS One. 2013 Dec 2;8(12):e82245. doi: 10.1371/journal.pone.0082245 (PMC3846816; doi:10.1371/journal.pone.0082245)
Supplement: Table S1 — Summary of Abbreviated Injury Scores for the head region (AIS-HR), adapted from: Association for the Advancement of Automotive Medicine (2001) The Abbreviated Injury Scale, 1990 Revision, Update 98. Barrington, IL: Association for the Advancement of Automotive Medicine. (PDF) [file pone.0082245.s001.pdf]

Table S1. Summary of Abbreviated Injury Scores for the head region (AIS-HR), adapted from: Association for the Advancement of Automotive Medicine (2001) The Abbreviated Injury Scale, 1990 Revision, Update 98. Barrington, IL: Association for the Advancement of Automotive Medicine.

Radiological Diagnosis	AIS-HR
**Skull fracture**	
Vault fracture (simple)	2
Vault fracture (complex or depressed)	3-4
Basilar fracture (simple)	3
Basilar fracture (complex)	4
**Cerebral contusion**	
Small (<30 cc)	3
Large (30-50 cc)	4
Extensive (>50 cc)	5
**Subdural haematoma**	
Small (<25cc or 1cm thick)	4
Large (>25cc or 1cm thick) or bilateral	5
**Extradural haematoma**	
Small (<25 cc or 1 cm thick)	4
Large (>25cc or 1 cm) or bilateral	5
